# When can we give general anesthesia to an infant with anticipated difficult airway management caused by facial vascular malformation?

**DOI:** 10.1186/s40981-017-0082-9

**Published:** 2017-04-04

**Authors:** Kumi Moriyama, Masanori Mitsuda, Masakazu Kurita, Mine Ozaki, Kiyoshi Moriyama, Tomoko Yorozu

**Affiliations:** 1 Department of Anesthesiology, Kyorin University Faculty of Medicine, 6-20-2 Shinkawa, Mitaka, Tokyo 181-8611 Japan; 2 Department of Plastic Surgery, Kyorin University Faculty of Medicine, 6-20-2 Shinkawa, Mitaka, Tokyo 181-8611 Japan

**Keywords:** Pediatric, Facial vascular malformation, Anticipated difficult airway management

## Abstract

A 13-month-old infant weighing 8.3 kg with a height of 72.3 cm visited our hospital for surgical resection of facial vascular malformation detected at birth. Because we anticipated the patient would have difficult airway management and massive perioperative bleeding, we postponed surgery to discuss the appropriate timing and general anesthesia approach with anesthesiologists at other institutions, while explaining the risk of general anesthesia and bleeding to the parents. When the patient was 21 months old and 10 kg, he started bleeding while undressing, when his lips touched his clothes. Because the cricothyroid membrane puncture kit (QuickTrach Child™ (VBM Medizintechnik GmbH, Sulz am Neckar, Germany)) can be used on infants weighing over 10 kg, we decided to give him general anesthesia. The infant was successfully intubated by Airwayscope™ and the lesion was surgically removed in accordance with the preoperative plan. The procedure took 65 min and created 8 g of bleeding. The infant had no postoperative bleeding or respiratory complications. There is no data on the timing of safe anesthesia management in infants with difficult airway management. Thus, taking the time to discuss the case with surgeons, other anesthesiologists, and the parents can be helpful.

## Background

To date, there is little clinical or experimental evidence to guide the management of the “can’t intubate, can’t oxygenate” (CICO) scenario in pediatric anesthesia particularly regarding the infant or neonate [1]. We describe here an infant with facial vascular malformation. In this case, surgery was postponed until the infant grew up to 10 kg, as we anticipated the patient to have difficult airway management.

## Case presentation

**Fig. 1:**
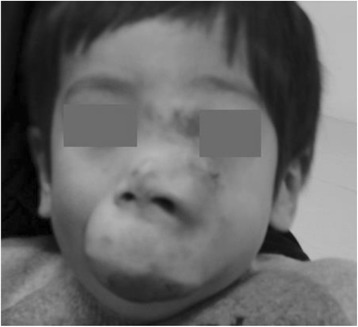
A 13-month-old infant weighing 8.3 kg, with a height of 72.3 cm. A large vascular malformation occupied his upper lip to his left face, and a swollen upper lip covered the lower lip

A 13-month-old infant weighing 8.3 kg with a height of 72.3 cm visited our hospital for surgical resection of facial vascular malformation detected at birth. Before the visit, he had visited another pediatric hospital, where a surgeon recommended that the infant’s parents postpone his surgery until school age. The patient experienced frequent spontaneous bleedings from the mucosal surface of his expanded upper lip, even without traumatic episodes. In order to reduce the frequency of the bleedings (as well as improving his appearance), the plastic surgeons in our hospital investigated the possibility of partial resection of the upper-lip vascular malformation to suppress bleeding from the lesion and consulted to our department. When the infant came to our preoperative management clinic, his upper left lip had a vascular malformation and his swollen upper lip covered the lower lip (Fig. 1). The vascular malformation was absent when we observed his oral cavity at vagitus. An adult face mask covered his face without pushing against the vascular malformation. The left ala of the nose also had vascular malformation, and the left nasal cavity easily collapsed when we put the mask on his face. In addition to the anticipated difficult airway management, massive perioperative bleeding was similarly expected. At this time point, we decided to postpone surgery to discuss the appropriate timing and approach to the use of general anesthesia, while explaining its risks to the parents, as well as his bleeding issues.

During this discussion, we confirmed that the cricothyroid membrane puncture kit (QuickTrach Child™ (VBM Medizintechnik GmbH, Sulz am Neckar, Germany)) was applicable to infants weighing over 10 kg. Although we had experience in surgical resection of vascular malformation in infants, we had little experience in the management of difficult airways in infants. Therefore, we recommended that the infant’s parents consult pediatric anesthesiologists at other pediatric hospitals, which do not perform surgical resection of infant facial vascular malformation. Two pediatric anesthesiologists suggested that intubation would be possible with tracheostomy and postoperative respiratory care.

At 20 months, the child was 9.6 kg and an otolaryngologist evaluated the laryngopharynx. On nasal endoscopic evaluation, no mucosal lesion was apparent in either nasal cavity or the pharynx and the larynx. We were unable to get good images via CT or MRI, as we usually do not give sedative drugs to infants with the possibility of upper airway invasion due to the vascular malformation. At 21 months, he was 10 kg and started bleeding while undressing when the lips touched the clothes; at this point, his hemoglobin was 6.8 g/dL. We suspected that induction of anesthesia would become more difficult when the facial vascular malformation grew larger, and decided that it was the appropriate time to give him general anesthesia.

On the operation day, pediatric surgeons gathered in the operating room in case of emergent surgical airway management. The infant entered the operating room without premedication and with a 24 G infusion route. General anesthesia was slowly induced with 2% sevoflurane and N_2_O in oxygen. Bag/mask ventilation was established, and 0.01 mg/kg of atropine, 4 μg/kg of fentanyl, and 0.1 mg/kg of midazolam were injected intravenously. We inserted an Airwayscope™ (Hoya, Tokyo, Japan) while spontaneously breathing with an additional bolus dose of 1.1 mg of midazolam. The vocal cord was visible using Airwayscope™ and a 4.0-mm endotracheal tube was easily inserted. The SpO_2_ was maintained from 97 to 100% during induction of anesthesia, and we administered 22 mg of rocuronium intravenously after the airway was secured. The lesion was surgically removed in accordance with our preoperative plan, taking 65 min with 8 g of bleeding. After confirming spontaneous respiration, 40 mg of sugammadex was intravenously infused and the endotracheal tube was removed. Because his body movement was intense after extubation, we gave him 0.05 mg/kg of midazolam and 1 μg/kg of fentanyl and then transferred him to the intensive care unit. Vascular malformation in his upper lip was removed, but the left ala of the nose still exhibited vascular malformation, so that the overall risk of massive nose bleeding was unchanged. The infant exhibited no bleeding or respiratory complications afterwards and was discharged on the 7th postoperative day. The parents were informed about the risk of massive nose bleeding if he fell down.

The patient was followed up at outpatient clinics for 4 months without any signs of bleeding, and the second surgery was planned 4 months later for further improvement of his appearance. However, the infant accidentally fell to the ground before this planned surgery and unfortunately died of hemorrhagic shock.

## Discussion

When facial vascular malformations are compromised with anticipated difficult airway management, it becomes more difficult to determine appropriate timing of surgery. Few case reports exist that describe airway management for surgical resection of facial hemangioma in children [2, 3]. Although they can have rapid progression and spontaneous involution in 90% of cases, vascular malformation usually shows slow progression, without spontaneous regression [4]. They also require different therapeutic management, depending on its localization; in this case, as the vascular malformation in his upper lip started bleeding while undressing, the first surgery was scheduled for resection of the vascular malformation in his upper lip. Although this surgery was safely performed as planned, the risk of massive nose bleeding eventually became lethal. It is difficult to ascertain the exact optimal time, since other factors like vascular malformation can influence the airway, risk of bleeding, heart load due to hypervolemia, and parental mental distress.

In the presented case, we decided that appropriate timing for general anesthesia in an infant with anticipated difficult airway management was when the infant grew to 10 kg and could be treated with the cricothyroid membrane puncture kit. Although it is difficult to evaluate the safety of cricothyrotomy devices for infants, Metterlein et al. reported successful placement of QuickTrach Baby™ (VBM Medizintechnik GmbH, Sulz am Neckar, Germany) in adult rabbits [4]. However, Stacey et al. reported that cannula tracheostomy in a model comparable to an infant’s airway was still difficult and not performed without complication [5]. Because little evidence exists to guide the management of the “can’t intubate, can’t oxygenate” scenario in pediatric anesthesia [1], weight may not predict appropriate timing for general anesthesia in an infant.

In this case, we did not use muscle relaxants during endotracheal intubation, although they are recommended in the management of unexpected difficult pediatric airways [6]. As stated, in this kind of pediatric airway management, this does not relate to failure to intubate, but failure to recognize and overcome functional airway problems; this is due to insufficient depth of anesthesia or muscle paralysis, leading to morbidity and mortality. In addition, sugammadex can immediately reverse the neuromuscular block caused by rocuronium or vecuronium [7]. Muscle paralysis would similarly help reduce the risk of accidental bleeding from the tumor, particularly at the upper lip. However, we did not use muscle relaxants, as we had little experience in the management of difficult infant airways. Therefore, in this case, we preferred to maintain spontaneous breathing, which is what we usually do when managing anticipated difficult airways in adults.

It would have been preferable if this surgery was performed at a children’s hospital with pediatric anesthesiologists, although we could not find any appropriate surgeons for the possibility of surgical removal, along with an acceptable volume of intraoperative bleeding. As a secondary measure, we encouraged the parents to talk with several anesthesiologists at children’s hospitals before treatment at our hospital. This helped the parents understand the infant’s pathology and the inherent risks associated with airway management; this understanding also alleviated their level of anxiety.

We did not decide whether to extubate in advance of surgery, so an endotracheal tube was successfully removed afterwards, with the infant immediately requiring sedative drugs. Because the vascular malformation was removed as planned, we interpreted it to mean that there was no further risk of airway obstruction by his vascular malformation. When resection of facial vascular malformation is planned, children are usually not cooperative enough to evaluate the degree of invasion of the vascular malformation. However, it becomes clear after induction of general anesthesia, whether the lesion can be securely and surgically removed then. It may be a safe strategy to use general anesthesia and not extubate all patients in the operating room, although prolonging unnecessary intubation can cause other problems in the ICU. The decision to extubate requires close communication with the surgeons, in accordance with the progress of the surgical procedure.

## Conclusions

In conclusion, we experienced anesthetic management of an infant who was compromised with facial vascular malformation. Because we were aware that the patient had anticipated difficult airway management, surgery was postponed until the infant grew to 10 kg. There is no data on the optimal timing of safe anesthesia management in infants with difficult airway management. Thus, it was helpful to have time to discuss this case with other surgeons and anesthesiologists, as well as the parents.
